# Genome-wide identification, expression, and sequence analysis of *CONSTANS-like* gene family in cannabis reveals a potential role in plant flowering time regulation

**DOI:** 10.1186/s12870-021-02913-x

**Published:** 2021-03-17

**Authors:** Gen Pan, Zheng Li, Ming Yin, Siqi Huang, Jie Tao, Anguo Chen, Jianjun Li, Huijuan Tang, Li Chang, Yong Deng, Defang Li, Lining Zhao

**Affiliations:** 1 Institute of Bast Fiber Crops, Chinese Academy of Agricultural Sciences, Changsha, 410205 China; 2 Key Laboratory of the Biology and Process of Bast Fiber Crops, Ministry of Agriculture, Changsha, China

**Keywords:** Cannabis, Genome-wide, *CONSTANS-like* gene (*COL*), Expression pattern, Flowering time

## Abstract

**Background:**

Cannabis, an important industrial crop, has a high sensitivity to photoperiods. The flowering time of cannabis is one of its important agronomic traits, and has a significant effect on its yield and quality. The *CONSTANS-like* (*COL*) gene plays a key role in the regulation of flowering in this plant. However, the specific roles of the *COL* gene family in cannabis are still unknown.

**Results:**

In this study, 13 *CsCOL* genes were identified in the cannabis genome. Phylogenetic analysis implied that the CsCOL proteins were divided into three subgroups, and each subgroup included conserved intron/exon structures and motifs. Chromosome distribution analysis showed that 13 *CsCOL* genes were unevenly distributed on 7 chromosomes, with chromosome 10 having the most *CsCOL* members. Collinearity analysis showed that two syntenic gene pairs of *CsCOL4* and *CsCOL11* were found in both rice and *Gossypium raimondii*. Of the 13 *CsCOL* genes, *CsCOL6* and *CsCOL12* were a pair of tandem duplicated genes, whereas *CsCOL8* and *CsCOL11* may have resulted from segmental duplication. Furthermore, tissue-specific expression showed that 10 *CsCOL* genes were preferentially expressed in the leaves, 1 *CsCOL* in the stem, and 2 *CsCOL* in the female flower. Most *CsCOL* exhibited a diurnal oscillation pattern under different light treatment. Additionally, sequence analysis showed that *CsCOL3* and *CsCOL7* exhibited amino acid differences among the early-flowering and late flowering cultivars.

**Conclusion:**

This study provided insight into the potential functions of *CsCOL* genes, and highlighted their roles in the regulation of flowering time in cannabis. Our results laid a foundation for the further elucidation of the functions of *COL* genes in cannabis.

**Supplementary Information:**

The online version contains supplementary material available at 10.1186/s12870-021-02913-x.

## Background

Hemp (*Cannabis sativa* L.) is an ancient economic crop that is widely used in textiles, food, and building materials, as well as other fields [1]. In recent years, the use of cannabidiols, represented by cannabinoid (CBD), has been expanding continuously, and the cannabis industry has demonstrated good prospects for development in the future [2]. Hemp is an annual short-day crop that is sensitive to photoperiods [3]. Cannabis cultivars naturally grow in high-latitude areas. However, cannabis germplasms have been introduced to low-latitude areas for planting, resulting in an early-flowering time. As a result, the growth period has been shortened, seriously reducing the yield and content of CBD and fibre [3]. Thus, the development of cannabis varieties with a wide adaptability is one of the main goals of current cannabis breeding programs. Identifying the regulatory mechanism of cannabis flowering could provide a theoretical foundation for the cultivation of cannabis varieties. However, studies on the regulatory mechanism of flowering in cannabis are currently lacking.

The flowering period of plants is a complex quantitative trait that is comprehensively regulated by many internal and external factors, including the photoperiod, temperature, hormones, and self-development [4]. Among these factors, the photoperiod is an important regulatory factor of the floral transition. In agriculture, the flowering time of cultivated plants can be adjusted to meet consumer demand by changing the length of exposure to light. With rapid advances in the fields of molecular genetics and molecular biology, many genes related to the photoperiod pathway have been discovered and cloned [5, 6]. Studies have shown that the *CONSTANS-like (COL)* genes are important regulators of the plant response to photoperiods and is a core element in the regulation of plant flowering [7–9]. *COL* belongs to the zinc finger transcription factor family, which contains a B-box-type and a CCT (CO, CO-LIKE, TOC1) domain [10]. Depending on the number of B-box and CCT domains, *COL* family genes can be divided into five groups [11]. In previous reports, the *COL* gene family has been comprehensively studied in many plants, including *Arabidopsis*, rice (*Oryza sativa* L.), maize (*Zea mays* L.), *Populus*, radish (*Raphanus sativus* L.), moso bamboo (*Phyllostachys heterocycla*), and *Lilium × formolongi* [5, 6, 10–14]. The number of *COL* genes varies among different species. For example, among dicot plants, the *COL* family has 20 members in radish and 17 members in *Arabidopsis*, while in monocots, 16 members have been identified in rice, 19 members in maize, and 14 members in *Populus* [12–16].

The *COL* gene functions as a transcription factor in multiple growth and development pathways, and particularly in the photoperiod-mediated flowering pathway. Some genes in this family have been found to play an important role in the light response-mediated regulation of flowering [5, 17–19], with functions that differ between short-day (SD) and long-day (LD) conditions. For example, *OsCOL10, OsCOL13,* and *OsCOL16* function as negative regulators of flowering under both SD and LD condition in rice, while *Hd1*, a member of the *COL* gene family, promotes flowering under SD and suppresses flowering under LD [5, 17, 20, 21]. In *Arabidopsis*, the overexpression of *AtCOL3*, *AtCOL7,* and *AtCOL8* can delay flowering time, while, in contrast, the overexpression of the *AtCOL5* gene promotes flowering by enhancing the expression of *FLOWERING LOCUS T* (*FT*) [18, 19, 22, 23]. Similar to their functions, the expression patterns also vary among the members of the *COL* gene family. In bananas (*Musa nana* Lour.), *MaCOL* genes display higher expression in light than in darkness, reaching their peak during light periods [24]. The transcript levels of *PaCOL1* and *PaCOL2*, 2 members of the *COL* gene family in Norway spruce (*Picea abies* L.), are induced by light and increase upon transition from darkness to light [25]. Unlike *PaCOL1* and *PaCOL2*, *PttCO1* and *PttCO2* showed a distinct expression pattern with an increase in expression in the early evening [26]. Meanwhile, the differences in the sequences of these genes in the CDS region were reported to associate with their functions in the photoperiod-mediated flowering pathway. For example, the deletion of 2 bp in the second exon of *Hd1* in “Kasalath” resulted in delay of flowering time in rice accessions [20]. Similarly, nucleotide polymorphisms in the *OsCOL16* coding sequence were mainly composed of three alleles (A1, A15, and A22), which varied with the flowering time [5]. These studies indicated that, due to differences in the expression patterns and CDS sequences, the *COL* gene family performs multiple functions in the regulation of flowering time under SD and LD conditions.

Although *COL* genes play an important role in the growth and development of many plants, a comprehensive analysis of the *COL* family genes in cannabis is currently lacking. In addition, no systemic analyses of any other gene families in cannabis have been conducted, due to the unavailability of cannabis genome assembly, with a lack of information on gene locations at the chromosome level. The genome of cannabis was recently sequenced and made available on the cannabis genomic database [27], allowing for a comprehensive analysis of the *COL* gene family in cannabis. In the present study, the *COL* gene family from cannabis was analysed using bioinformatics, and the temporal and spatial expression patterns of the *COL* gene were studied. Additionally, differences in the amino acid (aa) sequences of *CsCOL3* and *CsCOL7* between early- and late-flowering cultivars were explored. Thus, the results presented in this study provide a biological basis for further studies to analyse the molecular functions of the *CsCOL* gene family in cannabis.

## Results

### Identification of 13 *CsCOL* genes in cannabis

A total of 13 *CsCOL* genes were identified from the cannabis genome database (*CsCOL1* through *CsCOL13*). The 13 *CsCOL* genes included both B-box and CCT conserved domains. Their physicochemical properties were analysed using ProtParam (http://web.expasy.org/protparam/) (Table 1). As shown in Table 1, the lengths of CsCOL proteins varied from 184 (CsCOL8) to 507 (CsCOL12) aa, molecular weights ranged from 26.02 kDa to 56.24 kDa, and pI varied from 4.99 to 6.36. In addition, the grand average of hydropathicity varied from − 1.088 to − 0.245 and the aliphatic index ranged from 38.10 to 69.90 (Table 1).

**Table 1:** Characteristics of 13 CsCOL proteins in cannabis

Gene	Gene ID	Length (aa)	MW (Da)	pI	GRAVY	Aliphatic index
CsCOL1	LOC115722074	375	42,446.16	5.82	−0.766	61.63
CsCOL2	LOC115714019	462	51,911.74	5.42	−0.687	61.00
CsCOL3	LOC115697429	443	48,003.43	5.26	−0.563	61.87
CsCOL4	LOC115725326	387	41,576.35	5.08	−0.245	69.90
CsCOL5	LOC115714839	507	56,248.68	5.95	−0.752	60.95
CsCOL6	LOC115711648	456	52,718.08	5.52	−1.069	58.62
CsCOL7	LOC115707536	337	36,832.04	5.89	−0.555	64.04
CsCOL8	LOC115703699	184	20,459.75	4.30	−1.167	38.10
CsCOL9	LOC115700798	407	43,842.56	4.89	−0.489	60.96
CsCOL10	LOC115700744	394	42,808.34	5.30	−0.610	58.43
CsCOL11	LOC115700712	241	26,026.97	4.32	−0.741	55.85
CsCOL12	LOC115711183	420	48,419.95	5.23	−1.088	59.00
CsCOL13	LOC115700838	398	43,959.03	5.19	−0.513	58.84

*MW* molecular weight, *pI* isoelectric point

### Gene structure, phylogenetic relationship, and sequencing analysis of *CsCOL* genes

**Fig. 1:**
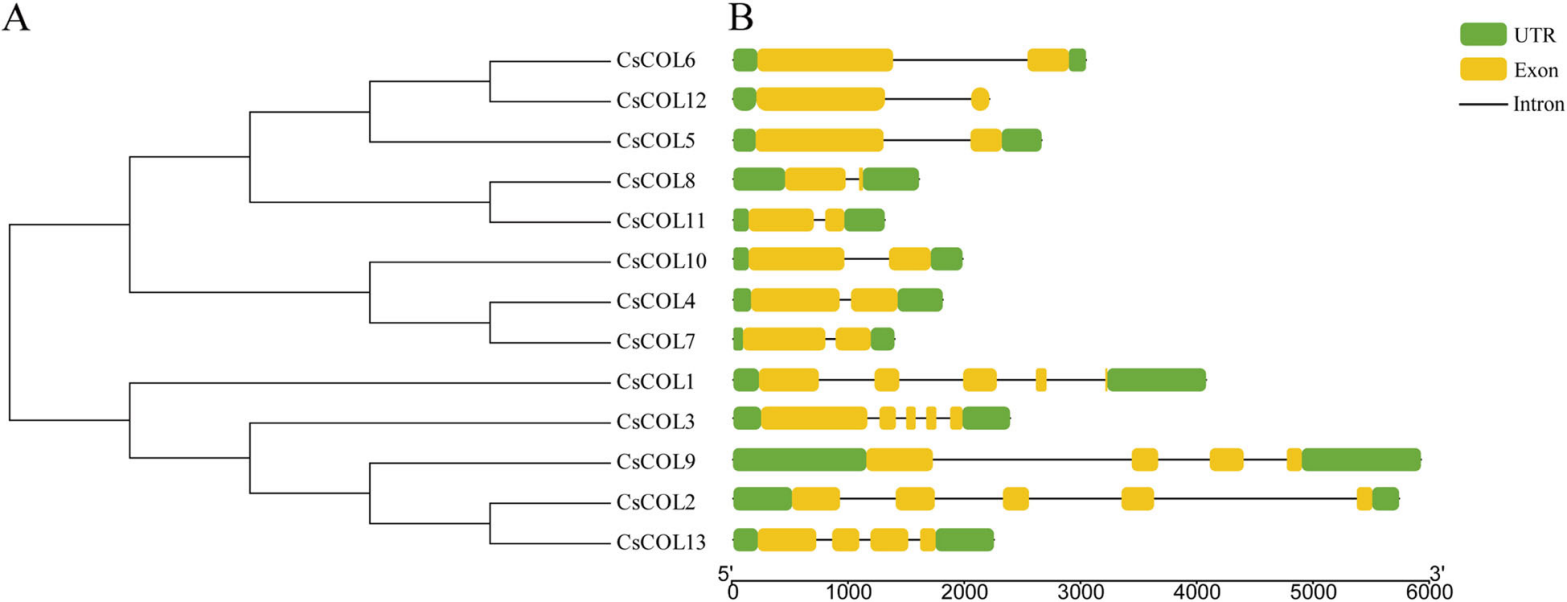
Phylogenetic and gene structure analyses of cannabis *CsCOL* genes. **a** Phylogenetic analysis. **b** Gene structures. The exon, untranslated region (UTR), and intron are represented by the yellow and green rectangles, and a black line, respectively

**Fig. 2:**
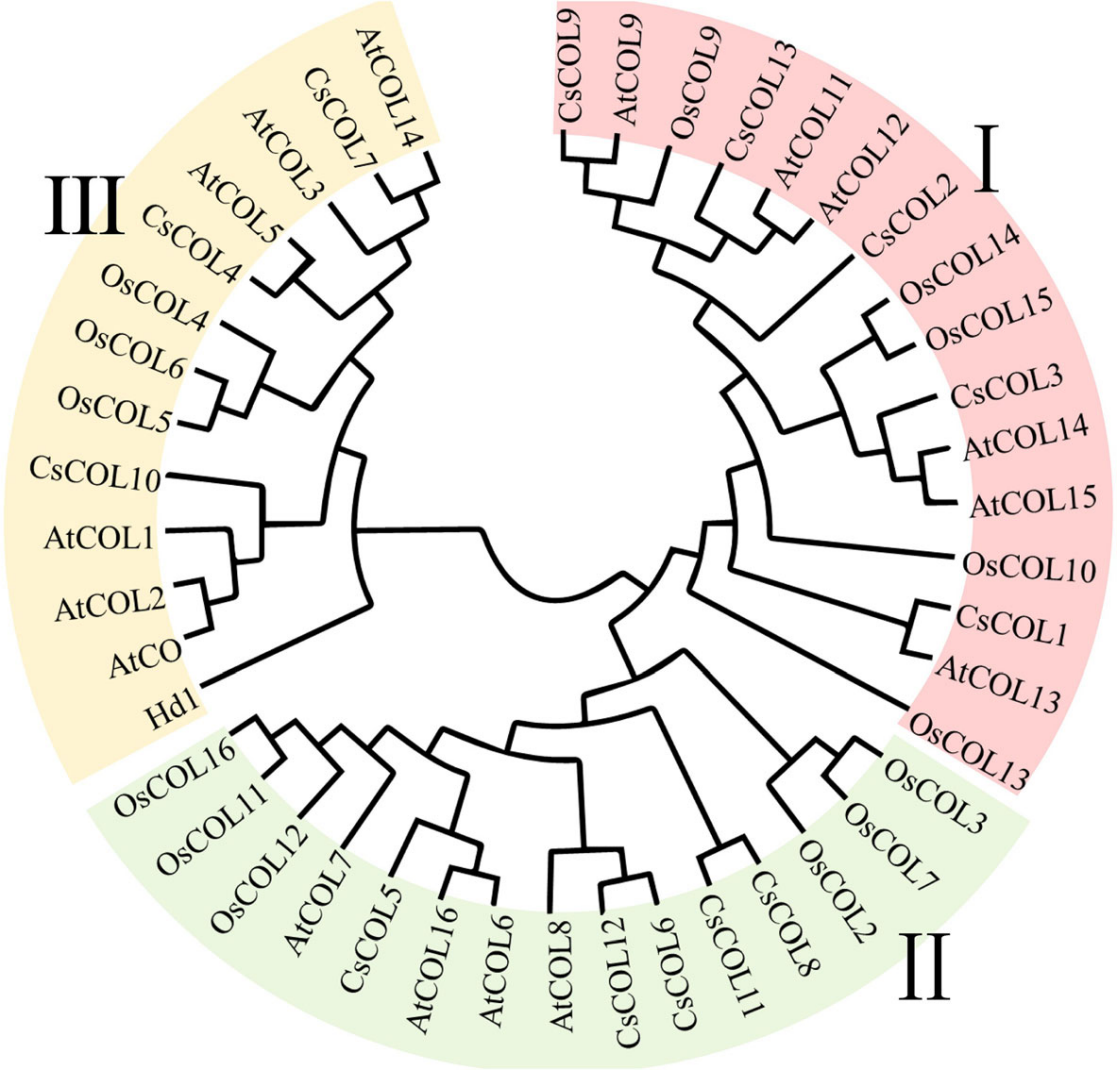
Phylogenetic tree of the COL proteins from three plant species. The phylogenetic tree was constructed based on the 90% shared amino acid sites using the neighbour-joining (NJ) method. At, *Arabidopsis thaliana*; Os, *Oryza sativa*; Cs, *Cannabis sativa*

To estimate the evolutionary relationships between the members of the *CsCOL* gene family, we investigated the structure diversity by comparing the gene structure of the CsCOL protein. As shown in Fig. 1, all the *CsCOL* genes contained 2 to 5 exons and 1 to 4 introns, respectively. All of the *CsCOL* genes contained 3′ and 5′ UTR regions (Fig. 1). Furthermore, to explore the evolutionary relationships between the *COL* genes of different species, phylogenetic tree analysis was conducted with COL proteins from different plants, including *Arabidopsis*, cannabis, and rice. These included 30 genes from dicotyledonous plants (e.g., *Arabidopsis* and cannabis) and 14 genes from monocotyledonous plants (e.g., rice). The results revealed that these COL proteins could be clustered into three major groups, named groups I–III (Fig. 2). Group III was the smallest subfamily, which was comprised of the lowest number of COL proteins (Fig. 2). In addition, we investigated the amino acid sequence of the CsCOL genes in “Y7” and “Q1”, a late- and an early-flowering varieties (Fig. S1 and Fig. S2). Unfortunately, only CsCOL3 and CsCOL7 were successfully cloned. For CsCOL3, 2 amino acid differences were found between “Y7” and “Q1”, neither of which was located in a B-Box nor CCT domain (Fig. S3A). With regards to CsCOL7, 4 amino acid differences were found between “Y7” and “Q1”, all of which were located in the B-BoxIdomain (Fig. S3B).

### Chromosomal location and synteny analysis

**Fig. 3:**
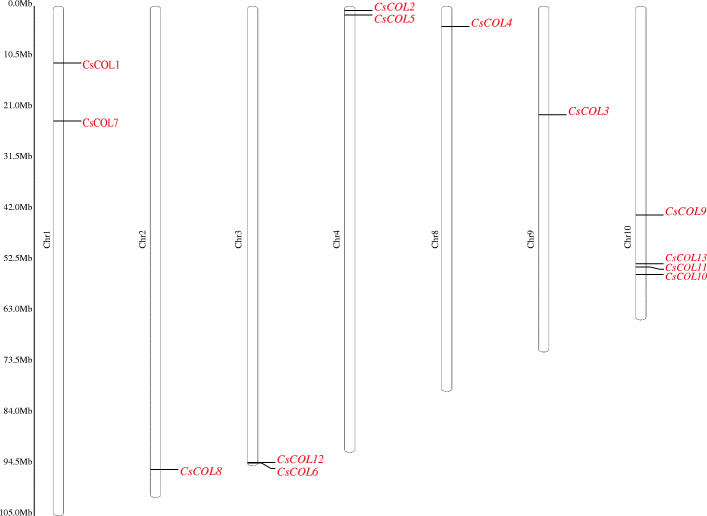
The physical location of 13 *CsCOL* genes on cannabis chromosomes. Chromosome numbers are indicated on the left of each scaffold. Chromosome size is shown by the vertical scale

**Fig. 4:**
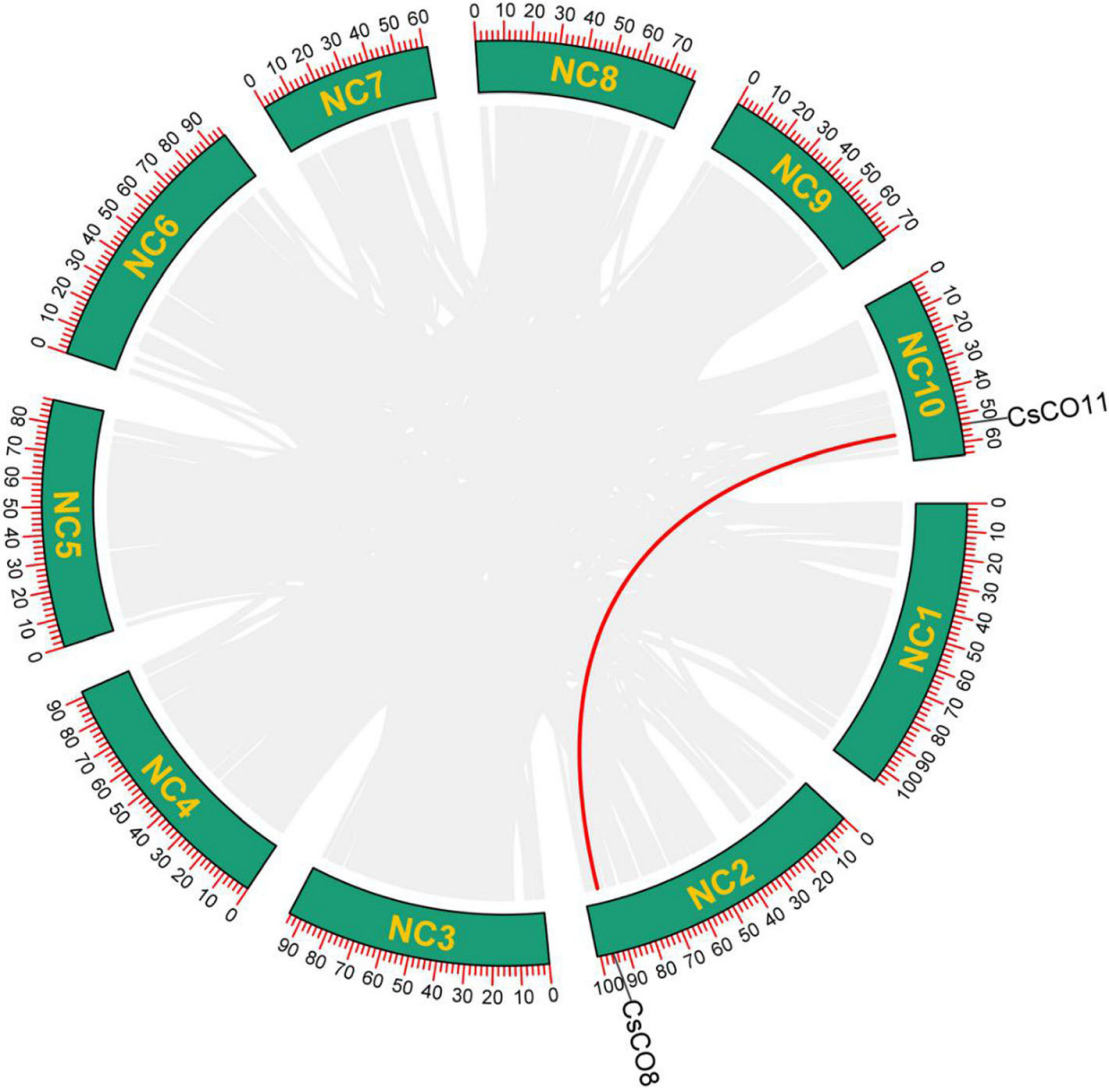
Schematic representation of the interchromosomal relationships between the *CsCOL* genes in the cannabis genome. Coloured lines indicates the colinear gene pair, grey lines indicate the syntenic blocks in the cannabis genome

**Fig. 5:**
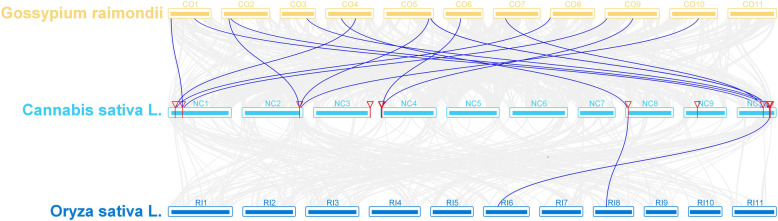
Synteny analysis of the *COL* genes between cannabis and 2 representative species. Coloured lines highlight the colinear gene pair, while grey lines indicate the syntenic blocks within cannabis and other plant genomes

As shown in Fig. 3, the 13 *CsCOL* gene members were found to be unevenly distributed across 7 chromosomes of the cannabis genome, except for chromosomes 5, 6, and 7. Among these, chromosome 10 had the highest number of *CsCOL* genes (4), while chromosomes 2, 8, and 9 only contained 1. Interestingly, a pair of tandem replication genes were identified in chromosome 3 (*CsCOL6/CsCOL12*), suggesting that tandem duplication events participated in the expansion of the *COL* family in cannabis. As such, duplication events were investigated for the *CsCOL* genes of the cannabis genome. As a result, only one pair of duplicated genes (*CsCOL8/CsCOL11*) was identified within the cannabis genome, they may have resulted from segmental duplication or whole genome duplication (WGD) (Fig. 4). In order to further understand the evolutionary mechanism of the *COL* family in cannabis, collinearity diagrams of the *COL* family were constructed in 2 dicotyledonous plants (*Gossypium raimondii* and *Cannabis sativa* L.) and 1 monocotyledonous plant (*Oryza sativa* L). As shown in Fig. 5, 15 pairs of orthologous genes were identified between cannabis and cotton Raymond, much greater than those identified between cannabis and rice (2). Among these genes, *CsCOL4* and *CsCOL11* were identified in both rice and cotton Raymond, *CsCOL1*, *CsCOL7*, *CsCOL8*, *CsCOL5*, and *CsCOL9* were found in cotton Raymond alone, and the remaining were not present in any of the duplicated blocks (Fig. 5).

### Spatial and temporal expression pattern analysis of 13 *CsCOL* genes

**Fig. 6:**
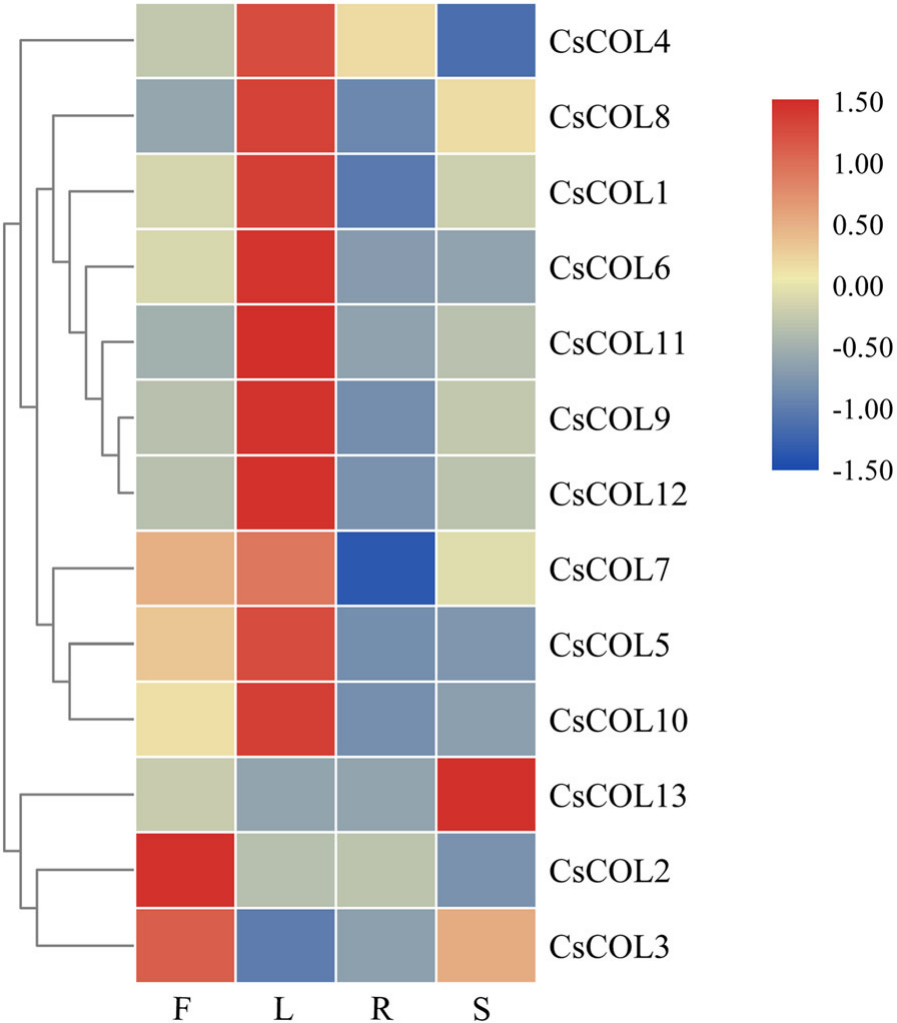
Tissue-specific gene expression of 13 *CsCOL* genes in cannabis. The scale bar indicates the logarithmic normalized expression level of each gene in different tissues. Red and blue indicate higher and lower transcript abundance, respectively. Three independent samples were used in expression analysis. F: female flower; L: leaf; R: root; S: stem

To gain insights into the possible role of *CsCOL* genes in the development of cannabis, the expression pattern of the *CsCOL* genes was analysed in 4 plant tissues: female flower, stem, leaf, and root. The results revealed that all genes were constructively expressed in various tissues, but with different expression patterns (Fig. 6). Among the 13 *CsCOL* genes, 10 were found to be highly expressed in the leaf tissue, *CsCOL2* and *CsCOL3* were highly expressed in the female flower, and different expression patterns were found for *CsCOL13*, with its highest expression level in the stem, and lower expression levels in other tissues (Fig. 6).

**Fig. 7:**
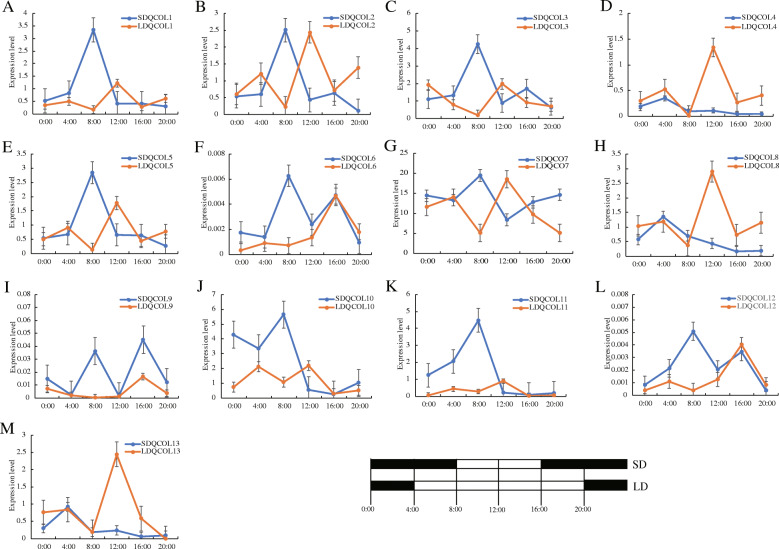
Expression patterns of the *CsCOL* genes under LD (8 h dark/16 h light) and SD (16 h dark/8 h light) conditions. SDQCOL1–13 represents the expression levels of *CsCOLs* of “Qingma 1” under SD conditions; LDQCOL1–13 represents the expression levels of *CsCOLs* of “Qingma 1” under LD conditions. Black rectangle represented the darkness, while white rectangle represented the light. Data shown as mean (±SD), *n* = 3

Previous studies found that the *COL* gene played an important role in the regulation of flowering time. To evaluate the possible functions of *CsCOL* genes, qRT-PCR was used to analyze the expression levels of *CsCOL* genes under different photoperiod treatments at 4 h intervals (Fig. 7). Under SD conditions, the diurnal expression pattern of the *CsCOL* genes varied. The expression patterns were roughly divided into three types (Fig. 7). The first type exhibited high levels of expression at the end of darkness, including *CsCOL1–3, CsCOL5–7* and *CsCOL10–12*. The second type showed an increased expression at 04:00 in the night (darkness), including *CsCOL4*, *CsCOL8* and *CsCOL13*. The remaining *CsCOL* genes displayed highest expression at the end of light (Fig. 7). Under LD conditions, although the transcript level of all *COL* genes was induced in light, two types of diurnal expression patterns were observed (Fig. 7). The first type included the transcript levels of most *CsCOL* genes, which peaked at 12:00 PM in the day (light) (*CsCOL1–5*, *CsCOL7*, *CsCOL8*, *CsCOL10*, *CsCOL11*, and *CsCOL13*), while the second category exhibited the highest expression levels at 16:00 in the day (light) (*CsCOL6*, *CsCOL9*, and *CsCOL12*). Collectively, these results suggest that the majority of the *CsCOL* genes exhibited a diurnal oscillation expression pattern under the SD and LD conditions.

**Fig. 8:**
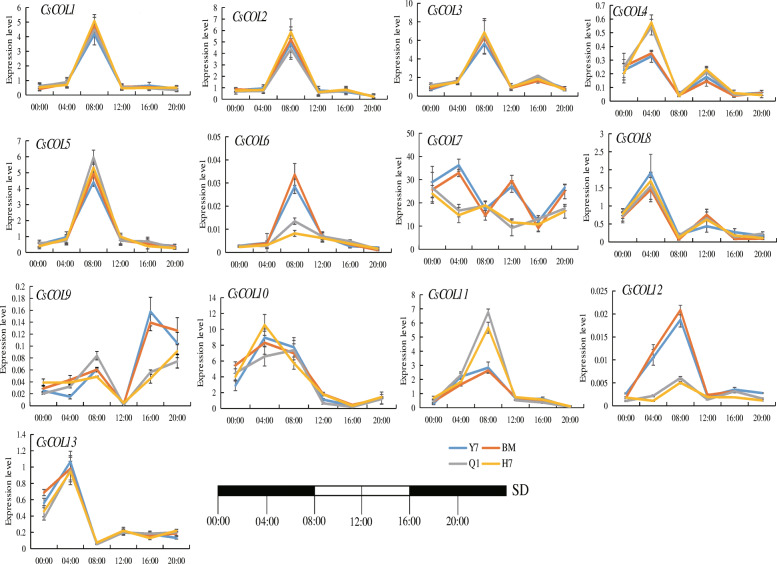
Expression patterns of the *CsCOL* genes of “Yunma 7” (“Y7”), “H7”, “BM”, and “Qingma 1” (“Q1”) under SD conditions (16 h dark/8 h light). Black rectangle represented the darkness, while white rectangle represented the light. Data shown as mean (±SD), *n* = 3

To further explore the function of *COL* genes in cannabis, 2 early-flowering varieties, “Qingma 1” (“Q1”) and “H7”, and 2 late-flowering varieties, “Yunma 7” (“Y7”) and “BM”, selected from 126 accessions growing under short days, were using for investigating the expression patterns of 13 *CsCOL* genes. The flowering time of “Q1” and “H7” is 29 d and 31 d after sowing, respectively, while those of “Y7” and “BM” is 117 d and 113 d, under SD conditions in the field (Fig. S1B). Under SD conditions, among the 13 *CsCOL* genes, 2 genes (*CsCOL4*, and *CsCOL11*) showed higher expression levels in these 2 early-flowering varieties than in these 2 late-flowering varieties at the peak of transcription levels, while 4 genes (*CsCOL6*, *CsCOL7*, *CsCOL9*, and *CsCOL12*) showed an opposing pattern. The remaining *COL* genes exhibited similar expression levels between these 4 varieties (Fig. 8).

## Discussion

The *COL* gene family plays a key role in the regulation of flowering time, and has been reported in many plant species, including *Arabidopsis*, rice, maize, soybean (*Glycine max*), tomato (*Solanum lycopersicum*), and *Populus*, *Lilium × formolongi* [5, 10, 12–14, 27, 28]. However, a genome-wide investigation of the *COL* family gene in cannabis has yet to be conducted. Due to the unavailability of a high-quality cannabis genome sequence, work on the genome-wide identification of *COL* genes in the cannabis genome has been lacking. The most recently assembled cannabis genome contained gene location information at the chromosome level [29], which allowed for a comprehensive analysis of the *COL* gene family in cannabis. In the present study, 13 *COL* members were identified in the cannabis genome. These were divided into 3 subgroups (Figs. 1 and 2), which is similar to the grouping in rice and *Arabidopsis* [16]. Similar numbers of *COL* genes were found in other plants, including *Populus* (14 *COL* genes), sorghum (*Sorghum bicolor* L.) (15), and rice (16) [13, 16, 30]. The fact that the size of the genome of the four plants differed suggests that the number of *COL* genes in the *COL* superfamily was stable and did not vary with genome size.

Tandem replication events are associated with the occurrence of novel functions and gene expansion. In cannabis, the replication events have been found to occur in the *CBCAS*, *THCAS*, and *CBDAS* genes [29, 31]. On the other hand, no tandem duplication events have been observed in the *COL* genes of cotton [30]. However, in this study, a tandem gene pair (*CsCOL6* and *CsCOL12*) was found on chromosome 3 in cannabis, indicating that gene replications may be an important driving force of cannabis gene evolution. In addition to tandem replication events, segmental duplication has been reported as the main driving force of gene expansion in the *COL-like* gene family in *Gossypium* and maize [12, 30]. Consistent with these findings, in this study, a segmental duplication gene pair (*CsCOL8/CsCOL11*) was found in the cannabis genome (Fig. 4). However, among the genes involved in duplication, this pair of duplication genes displayed different expression patterns under SD and LD conditions (Figs. 1, 7, and 8), which indicated that these genes experienced functional divergence during gene duplication. In addition, 15 pairs of orthologous genes between cannabis and cotton Raymond were identified, while only 2 pairs were found between cannabis and rice (Fig. 5). This observation implies that cannabis *COL* genes have a closer relationship with cotton Raymond than with rice, which may be consistent with the evolutionary relationship between monocotyledons and dicotyledons. Interestingly, *CsCOL4* and *CsCOL11* were found in both rice and cotton Raymond, indicating that these *COL* genes expanded in a species-specific manner from common ancestral genes before the dicot–monocot divergence.

Although previous studies have shown that *COL* genes are widely expressed in different plant tissues, they have been found to be preferentially expressed in the leaves [5, 14, 17]. Leaves sense photoperiod signals and express *COL* to activate FT and promote flowering [9]. In this study, we investigated the transcript levels of 13 *CsCOL* genes in various plant organs, including the female flower, leaves, roots, and stems. As a result, 10 *COL* genes in cannabis were found to be preferentially expressed in the leaves, with an expression pattern similar to that observed in other plants, indicating their potential functions (Fig. 7).

The photoperiod is considered to be a key determining factor of flowering timing in plants, and *COL* genes have been demonstrated to be involved in the regulation of photoperiod-mediated flowering [5, 9, 17]. Therefore, we investigated the diurnal variations in the transcript levels of the *CsCOL* genes under LD and SD conditions. Under SD conditions, all the *COL* genes exhibited a diurnal oscillation expression pattern, with few differences between them. The transcript levels of 9 *COL* gene members were found to peak at dawn (Fig. 7), similar to *OsCOL16*, *PtCOL1/2*, *AtCOL1*, *AtCOL2*, and *AtCO* in other plants [5, 13]. Under LD conditions, the expression patterns of all *CsCOL* genes were roughly divided into 2 types. The first type included 10 *CsCOL* genes that were expressed more highly after light treatment, peaking at 12 h, consistent with *COL* genes including *PtCOL14* in *Populus* [13]. Similar to *LfCOL13–16*, *OsCOL10*, and *OsCOL16*, the remaining 3 *CsCOL* genes showed higher expression levels in light than in darkness, and peaked at 16 h [5, 14, 17].

Different expression levels of *COL* genes may be associated with the flowering time in different varieties. In this study, the “Q1” and “H7” variety exhibited an earlier flowering time than “Y7” and “BM” under SD conditions in the field (Fig. S1B). To further determine the potential functions of *CsCOL* genes in the regulation of flowering time, we evaluated the transcript levels of all *CsCOL* genes in the “Q1”、“H7”、 “BM” and “Y7” varieties under SD conditions. As shown in Fig. 8, the expression levels of *CsCOL4* and *CsCOL8* were higher in the two early-flowering varieties (“Q1” and “H7”) than the two late-flowering (“Y7” and “BM”) at the peak transcript level, while six genes (*CsCOL6*, *CsCOL7*, *CsCOL9*, and *CsCOL12*) showed a contrasting pattern (Fig. 8). Interestingly, except for the similar expression patterns of *CsCOL6*, *CsCOL12*, and *OsCOL16* under SD and LD conditions (Fig. 7) [13], these 3 genes also belonged to the same subgroup based on their phylogenetic relationship analysis (Fig. 2). In a previous study, *CsCOL6* was found to repress flowering in rice [5]. Thus, *CsCOL6/CsCOL12* may exert a similar function to *OsCOL16* in cannabis. However, this requires further study.

Previous studies have suggested that differences in the amino acid sequences of *COL* genes could explain their varied functions in the photoperiod-mediated flowering pathway [5, 10, 20]. In the present study, differences in the amino acid sequences of *CsCOL3* and *CsCOL7* were observed between “Q1” and “Y7”, an early- and a late-flowering variety, respectively (Fig. S1 and Fig. S2). Moreover, differences were observed in the amino acid sequence of *CsCOL7* within the B-box, a conserved domain known for its functions in protein–protein interactions. However, whether these changes affect this type of function will need to be studied further.

## Conclusions

To summarize, this study is the first to provide a comprehensive analysis of the *COL* gene family in cannabis. Our aim was to elucidate the evolution, expression profiles, and potential functions of these genes in the regulation of flowering in cannabis. Although the possible functions of the *CsCOL* gene family require further study for validation, the systemic analysis conducted in this study provides a foundation for future studies on the biological and molecular functions of *COL* genes in cannabis.

## Methods

### Identification and analysis of physical and chemical properties of *CsCOL* gene family members in cannabis

The sequences of 17 *Arabidopsis* CONSTANS-like proteins were downloaded from the *Arabidopsis* Information Resource (TAIR) (http://www.aabidopsis.org/). The cannabis genome file and genome annotation file (assembly number: GCA_900626175.2) were obtained from the NCBI database (https://www.ncbi.nlm.nih.gov/) [29]. The software TBtools was used to compare the *Arabidopsis COL* gene with the cannabis genome by blast sequence alignment (E-value <1E^− 5^) and to screen the *CsCOL* family candidate genes in the cannabis genome. Next, the candidate genes were submitted to the Uniprot database (https://www.uniprot.org/) for batch comparison to verify whether they contained both CCT and B-box conserved domains. ProtParam (http://web.expasy.org/protparam/) was used to analyse various physicochemical parameters of the *CsCOL* genes.

### Gene cloning

The primers pairs of the *CsCOL* genes were designed according to the CDS sequences (Supplementary Table S1). cDNA of “Y7” and “Q1” was used as a template for each gene. PCR was performed as follows: an initial step at 94 °C for 5 min, followed by 30 cycles of 30 s at 98 °C, 30 s at 55 °C, 2 min at 68 °C, and a final extension of 10 min at 68 °C. After the PCR procedure was finished, the PCR product was purified, ligated to pGEM-T Easy vector, and transformed into *E. coli* DH5a. Positive clones were selected for sequencing. A list of the primers used for gene cloning is provided in Supplementary Table S1.

### Multisequence alignment, phylogenetic analysis of CsCOL proteins, and gene structure analysis of *CsCOL*

Multisequence alignment analysis of *Arabidopsis*, rice, and cannabis COL proteins was performed using Clustal X2.1 with the default parameters [32]. A phylogenetic tree was constructed using MEGA7.0 using the neighbour-joining (NJ) method. Bootstrap values (> 50%) were estimated using 1000 replicates. FigTree software was use to edit the phylogenetic tree. The protein structure of CsCOL was predicted using NCBI-CDD software online (https://www.ncbi.nlm.nih.gov/cdd/) with the default parameters (E-value < 0.01). The conserved motif (Motif) of the *CsCOL* genes was analysed using MEME software online (http://meme-suite.org), and the predicted number was set to 10. The coding sequence (CDS) and untranslated region (UTR) of *CsCOL* were extracted from the cannabis genome annotation file using TBtools, which was also used to combine evolutionary tree, gene conservative motif, CDS, and UTR to construct a diagram to compare the evolutionary relationships and structures of *CsCOL*.

### Chromosome distribution and synteny analysis of *CsCOL*

Information on the chromosome location of the *CsCOL* genes was extracted from the cannabis genome file and gene annotation file using TBtools. Next, the physical location of *CsCOL* genes on chromosomes was constructed using TBtools. TBtools, MCscanX, and Circos were used to calculate and draw the tandem repeats of *COL* on the chromosome, the collinear genes among the cannabis genome, and among different species.

### Evaluation of flowering time and photoperiod treatment

“Yunma 7” (“Y7”), “BM”, “H7”, and“Qingma 1” (“Q1”) were collected from the Institute of Bast Fibre Crops, China Academy of Agriculture Science, Changsha, China. The 2 varieties were randomly planted under natural short-day conditions in Changsha (southern China, 112°58′ E/28°11′ N, day length < 12 h during vegetative period). Once over 50% of the plants of each cultivar had bloomed, the flowering time was scored. For different photoperiod treatments under LD (16 h light/8 h dark) and SD (8 h light/16 h dark) conditions, the leaves of these seedlings were collected at 0:00, 04:00, 08:00, 12:00, 16:00, and 20:00 after photoperiod treatment. The resulting materials were promptly transferred into liquid nitrogen for RNA extraction, repeated independently in triplicate.

### RNA extraction and qRT-PCR analysis

Total RNA was extracted from various tissues and leaves under different photoperiods using an RNAprep Pure Plant Kit (Tiangen, Beijing). The cDNA was synthesizing using a PrimeScript 1st Strand cDNA Synthesis Kit (TaKaRa, Japan). According to the manufacturer’s instructions, quantitative RT-PCR (qRT-PCR) was conducted using a SYBR Premix Ex TaqTM kit (TaKaRa) on a 7500 Sequence Detection System (Applied Biosystems, USA). The DHS2 gene was amplified as the internal control. The primers used for qRT-PCR analysis are listed in Supplementary Table S2. The experiment was performed in triplicate.

## Supplementary Information

**Additional file 1: Table S1**. The primers used for gene cloning in this study

**Additional file 2: Table S2**. The primers used for qRT-PCR in this study

**Additional file 3: Fig. S1**. Comparison of flowering time between “Qingma 1”, “Yunma 7”, “H7”, and “BM”

**Additional file 4: Fig. S2**. Coning of *CsCOL3* and *CsCOL7* from “Yunma 7”(“Y7”) and“Qingma 1”(“Q1”)

**Additional file 5: Fig. S3**. Comparison of the amino acid sequences of CsCOL3 (A) and CsCOL7 (B) between “Yunma 7 (Y7)” and “Qingma 1 (Q1)”

## Data Availability

The datasets for supporting the conclusions of this article are listed in the article and its additional files. All coding sequences of *CsCOL3* and *CsCOL7* in “Qingma1” and “Yunma7” has been uploaded to the NCBI SRA database. SRA accession: PRJNA704531. The data will be accessible with the following link: “https://www.ncbi.nlm.nih.gov/sra/PRJNA704531”.

## Abbreviations

COL: CONSTANT-like
CBD: Cannabinoid
LD: Long day
SD: Short day
FT: FLOWERING LOCUS T
WGD: Whole genome duplication
CBCAS: CBCA synthases
THCAS: THCA synthases
CBDAS: CBDA synthases
qRT-PCR: quantitative real-time PCR
