# Supplementary material for: Genome-wide identification, expression, and sequence analysis of CONSTANS-like gene family in cannabis reveals a potential role in plant flowering time regulation
Source: BMC Plant Biol. 2021 Mar 17;21:142. doi: 10.1186/s12870-021-02913-x (PMC7972231; doi:10.1186/s12870-021-02913-x)
Supplement: Supplementary file 1 — Additional file 1: Table S1. The primers used for gene cloning in this study [file 12870_2021_2913_MOESM1_ESM.doc]

| **Gene** | **Forward primer** | **Reverse primer** |
| --- | --- | --- |
| *CsCOL3* | CTTTTCTGGCATTACTACGA | TGGTTCTTCATTAACCCTCA |
| *CsCOL7* | AAATCAGCTATGGCGTCGAA | TTTTCTTTCTCATTTTCTCGGT |
