# Supplementary figures and images for: Genome-wide identification, expression, and sequence analysis of CONSTANS-like gene family in cannabis reveals a potential role in plant flowering time regulation
Source: BMC Plant Biol. 2021 Mar 17;21:142. doi: 10.1186/s12870-021-02913-x (PMC7972231; doi:10.1186/s12870-021-02913-x)

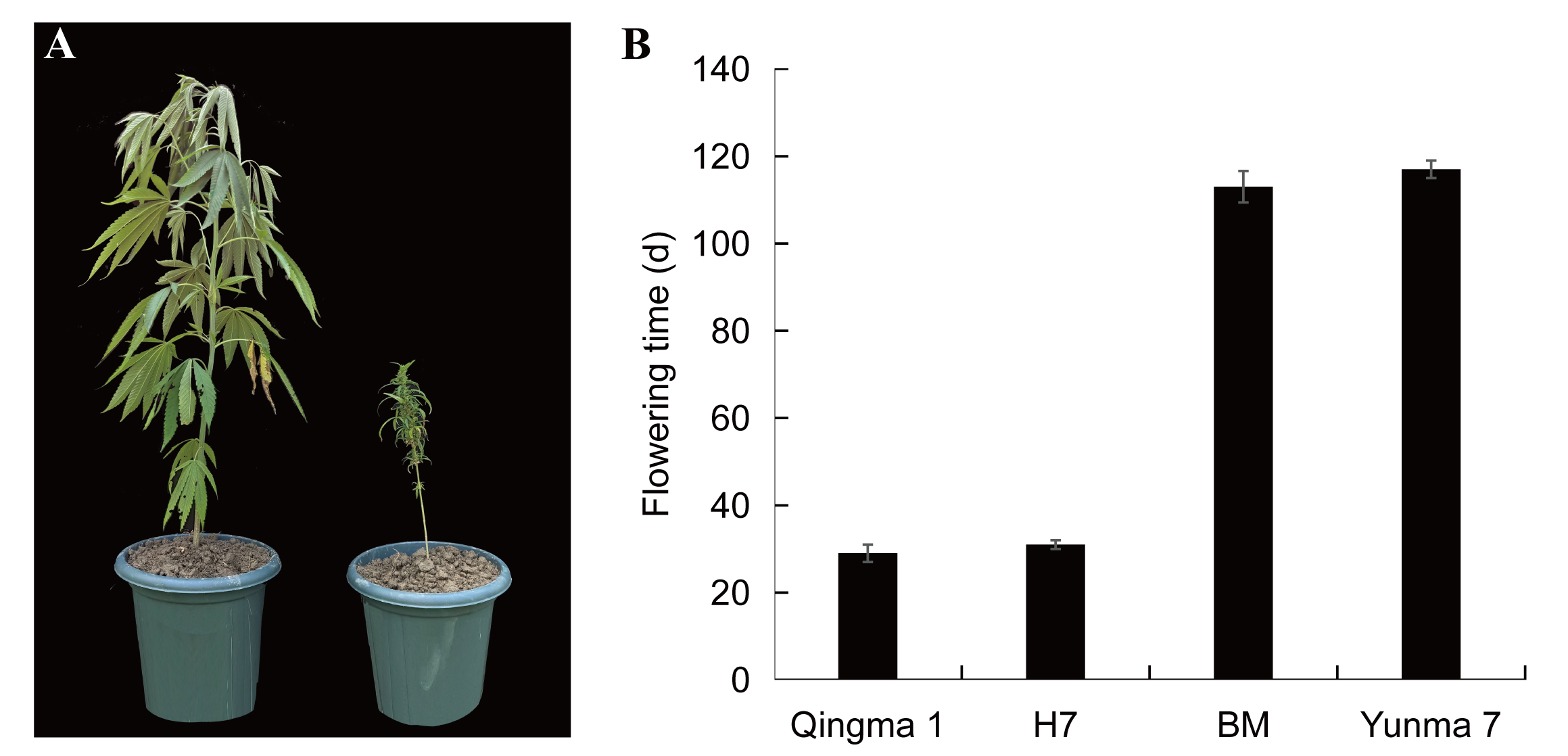

Supplement: Supplementary file 3 — Additional file 3: Fig. S1. Comparison of flowering time between “Qingma 1”, “Yunma 7”, “H7”, and “BM” [file 12870_2021_2913_MOESM3_ESM.jpg]

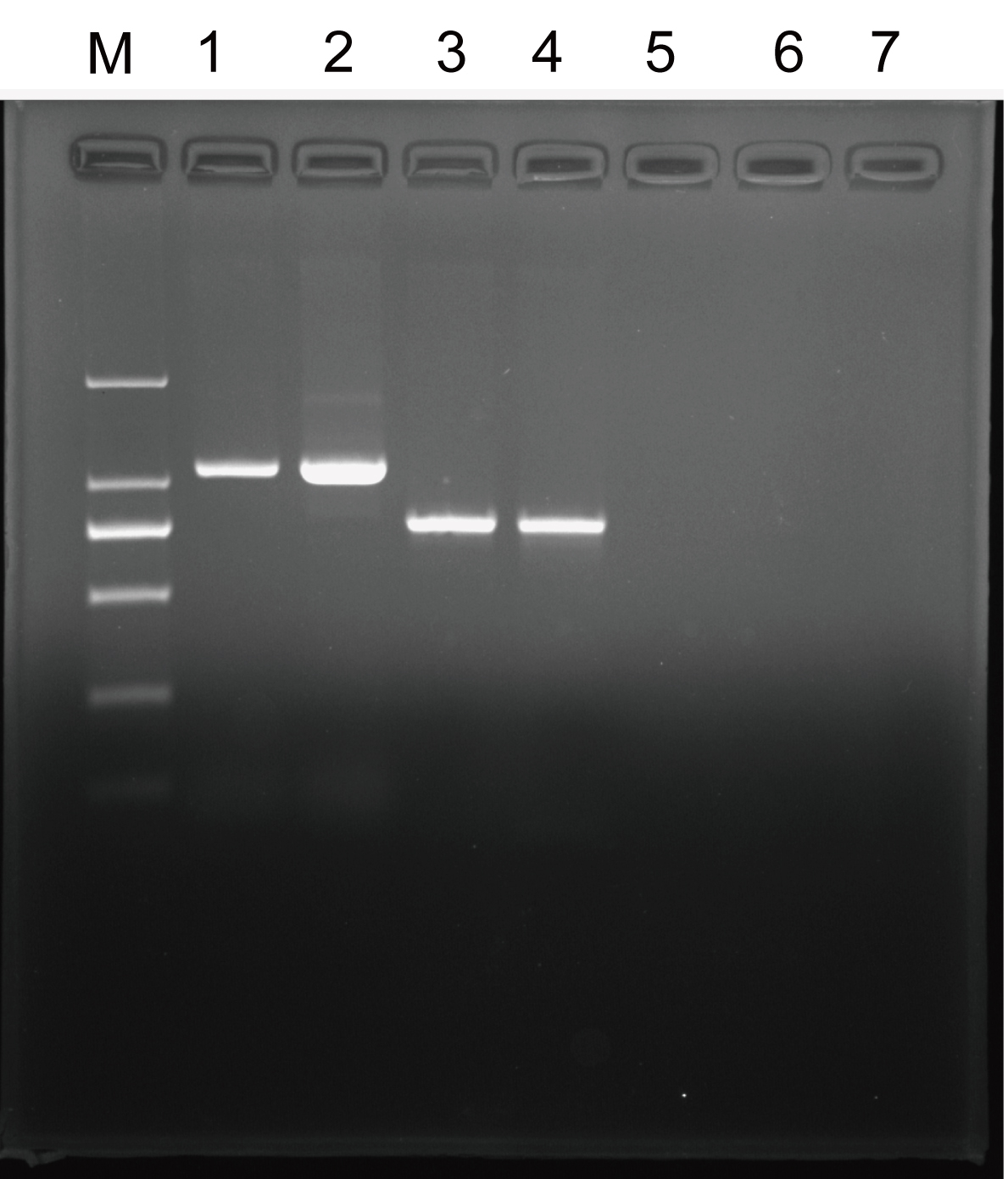

Supplement: Supplementary file 4 — Additional file 4: Fig. S2. Coning of CsCOL3 and CsCOL7 from “Yunma 7”(“Y7”) and“Qingma 1”(“Q1”) [file 12870_2021_2913_MOESM4_ESM.jpg]

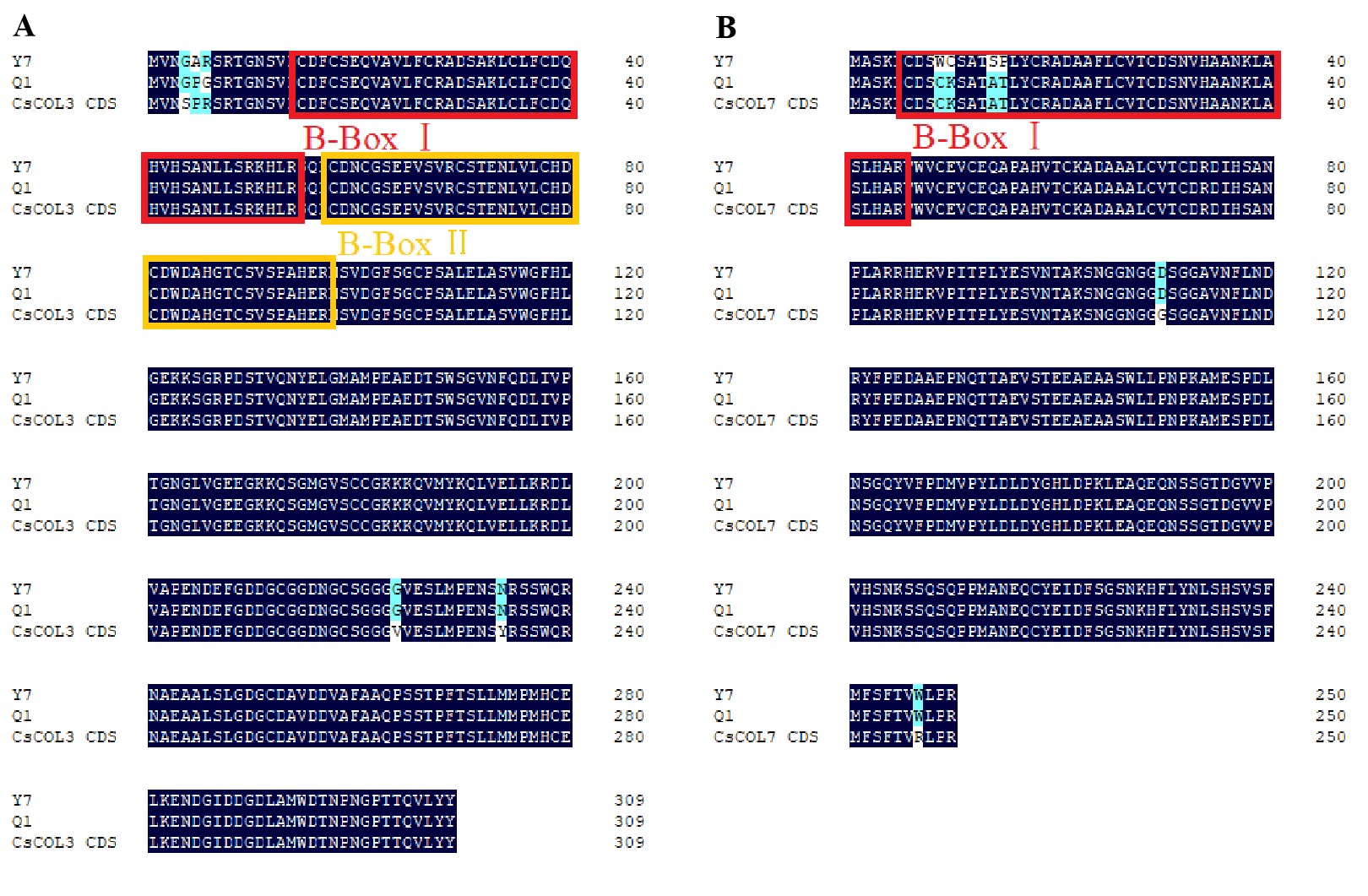

Supplement: Supplementary file 5 — Additional file 5: Fig. S3. Comparison of the amino acid sequences of CsCOL3 (A) and CsCOL7 (B) between “Yunma 7 (Y7)” and “Qingma 1 (Q1)” [file 12870_2021_2913_MOESM5_ESM.jpg]
