# Supplementary material for: Genome-wide identification, expression, and sequence analysis of CONSTANS-like gene family in cannabis reveals a potential role in plant flowering time regulation
Source: BMC Plant Biol. 2021 Mar 17;21:142. doi: 10.1186/s12870-021-02913-x (PMC7972231; doi:10.1186/s12870-021-02913-x)
Supplement: Supplementary file 3 [file 12870_2021_2913_MOESM2_ESM.doc]

Gene	Forward primer	Reverse primer
DHS2	TATCAAGGCTGTTCGTGGAG	AGGTGCTTTGATGGTGTTCC
CsCOL1	CTCTATCGTCCGCTCCTCTG	CGCCACCATGAGCTTAGAAT
CsCOL2	CAGAACCCCCAGAAGCAATA	GCTCAACTGCAAATGGATCA
CsCOL3	CGTCTGGGTTTGCGAAGTAT	CCGTTAGATTTTGCCGTGTT
CsCOL4	TGAGATGCGCAGAAGAGAGA	CCCCCAGTAGGGGAATCTAA
CsCOL5	AAGCCAGAACCCCTAGAAGC	GCTCAACTGCAAATGGATCA
CsCOL6	GCAGTTGAGCTTTGTAATCC	ACCCTAGAACTTCGTGTTCA
CsCOL7	AAGCCACAGTGTATCGTCTT	GCATATCGAATCGTCTTCTC
CsCOL8	AACTGTGTTAATGGCTGCTA	TGGTGGAGGTGGATTATTTG
CsCOL9	AGATTAATGCTGCATCCCAA	AAATTCAGGTCCACCTCATC
CsCOL10	GAGTATTTGGATCTGGTGGA	GAGAAACACCGTAACTCTGC
CsCOL11	AAGACGGTGATAATCAGGTG	ATGATCCAGATTGTCCTGAG
CsCOL12	TTTGCAGTTGAGCTTTGTAA	ACCCTAGAACTTCGTGTTCA
CsCOL13	GCGTAAAGGGTAGATTTGTG	AGGAAATGGTGTGAATGAAG
